# Selectively manipulable acoustic-powered microswimmers

**DOI:** 10.1038/srep09744

**Published:** 2015-05-20

**Authors:** Daniel Ahmed, Mengqian Lu, Amir Nourhani, Paul E. Lammert, Zak Stratton, Hari S. Muddana, Vincent H. Crespi, Tony Jun Huang

**Affiliations:** 1 Department of Engineering Science and Mechanics, The Pennsylvania State University, University Park, Pennsylvania 16802, USA; 2 Department of Physics, The Pennsylvania State University, University Park, Pennsylvania 16802, USA; 3 Department of Biomedical Engineering, The Pennsylvania State University, University Park, Pennsylvania, 16802 USA; 4 Department of Materials Science and Engineering, The Pennsylvania State University, University Park, PA 16802, USA; 5 Department of Chemistry, The Pennsylvania State University, University Park, Pennsylvania 16802, USA

## Abstract

Selective actuation of a single microswimmer from within a diverse group would be a first step toward collaborative guided action by a group of swimmers. Here we describe a new class of microswimmer that accomplishes this goal. Our swimmer design overcomes the commonly-held design paradigm that microswimmers must use *non-reciprocal* motion to achieve propulsion; instead, the swimmer is propelled by oscillatory motion of an air bubble trapped within the swimmer's polymer body. This oscillatory motion is driven by the application of a low-power acoustic field, which is biocompatible with biological samples and with the ambient liquid. This acoustically-powered microswimmer accomplishes controllable and rapid translational and rotational motion, even in highly viscous liquids (with viscosity 6,000 times higher than that of water). And by using a group of swimmers each with a unique bubble size (and resulting unique resonance frequencies), selective actuation of a single swimmer from among the group can be readily achieved.

The physics of swimming at the microscale^1^, where viscous forces dominate over inertial effects, is distinct from that at the macroscale^2^,^3^. Devices capable of finely controlled motion at the microscale could enable bold ideas such as targeted drug delivery^4^,^5^, non-invasive microsurgery^6^,^7^, and precise materials assembly^8^,^9^,^10^,^11^. Artificial microswimmers and nanomotors have been intensively developed over the past decade in an attempt to achieve controlled, powered, autonomous motion at the micro- and nanoscales^12^,^13^,^14^. Autonomous swimmers need to harvest energy from their environment and transduce it to mechanical form. For example, chemical energy can be harvested by bimetallic nanomotors that move by self-electrophoresis, decomposing a fuel such as hydrogen peroxide asymmetrically over their surfaces^15^,^16^,^17^,^18^,^19^. Motion can be modulated by chemical or optical gradients^20^, local analyte concentrations^21^, or local electrochemical control^22^. Micron-scale swimmers also can be powered, assembled, or steered by magnetic fields^23^,^24^,^25^,^26^,^27^, electric fields^28^, optical excitation^29^, acoustic scattering^30^,^31^,^32^,^33^,^34^, or thermal gradients^35^. Catalytically driven propulsion within more complex, confined reaction geometries is also possible^36^,^37^, as is generation of motion from the reorganization of hydrophobic/hydrophilic interactions within mobile microporous hosts^38^, or through quasi-oscillatory bursting at smaller length scales^39^.

Autonomous microswimmers are particularly compelling in biological or biomedical contexts. However, propulsion in a complex fluid medium (*i.e.*, highly viscous or non-Newtonian fluids) and motion against high flow rates has remained a significant challenge. In addition, many of the existing swimmers that use electric fields or chemical/electrochemical fuels cannot be used in biological environments such as the human body. More importantly, the prevailing goal of *selective actuation* of a single microswimmer from within a group—the first step towards collaborative action by a group of swimmers—has so far not been achieved. Here we demonstrate a new class of acoustic microswimmers that move through aqueous solution driven by ultrasonically powered bubble oscillation engines. These acoustic microswimmers respond to ambient acoustic energy, and do not need to gather chemical fuel from their environment. The means of implementing this propulsion mechanism are extremely simple and the applied acoustic fields are in the similar power intensity range as those used in ultrasonic imaging, which has proven to be a highly biocompatible, gentle technique. Furthermore, by creating bubbles of different sizes (and different resonance frequencies), selective actuation of a single swimmer from among the group can readily be achieved—a first in the field.

## Results

### Fabrication of the acoustic microswimmer

**Figure 1:**
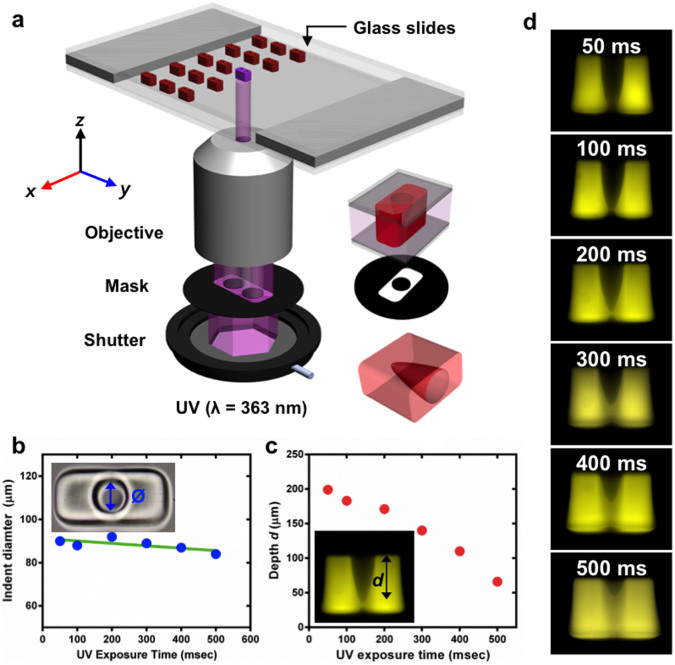
Fabrication and design of microswimmers. (a), Schematic of the fabrication setup. PEG solution containing photosensitive initiator is sandwiched between glass slides. The swimmers' geometries and the conical shaped indents were created by exposing the oligomer solution to UV light passing through a mask containing the blueprint of the swimmers. (b), Indentation diameter versus UV exposure time. (c), Indentation depth versus UV exposure time. (d), Images showing the decrease in indentation depth for increasing UV exposure time.

We fabricate the microswimmer using straightforward ultraviolet photopolymerization followed by chemical treatment to make the polymer surfaces hydrophobic (see Methods). The PEG/photo-initiator mixture was sandwiched between two glass slides; these slides were coated with PDMS to enable easy removal of the swimmer bodies once cured. The two slides were separated by 150 or 250 µm spacers, which determined the length of the swimmer body. Photomasks of different geometries (designed with AutoCAD software) were printed at 20,000 dpi resolution (CAD/Art Services, California). The mask was then inserted to the field stop of an inverted microscope (Nikon TE-2000U). A mercury lamp was used as the UV light source. A filter cube (11000v2: UV, Chroma) selected light of 373 µm wavelength. A shutter system, controlled by NIS software, adjusted the duration of UV exposure. The polymerization setup is shown schematically in Fig. 1a. The indentation diameter was controlled by the photomask, with a small amount of variability introduced by the UV exposure duration, as illustrated in Fig. 1b. Indentation depth was controlled by the UV exposure duration; the depth decreases with longer UV exposure, as illustrated in Fig. 1c. Conical indentation was due to defocusing of UV light across the thickness of the PEG/photoinitiator mixture, which leads to polymerization of the surrounding mixture.

### Mechanism of the acoustic microswimmer

**Figure 2:**
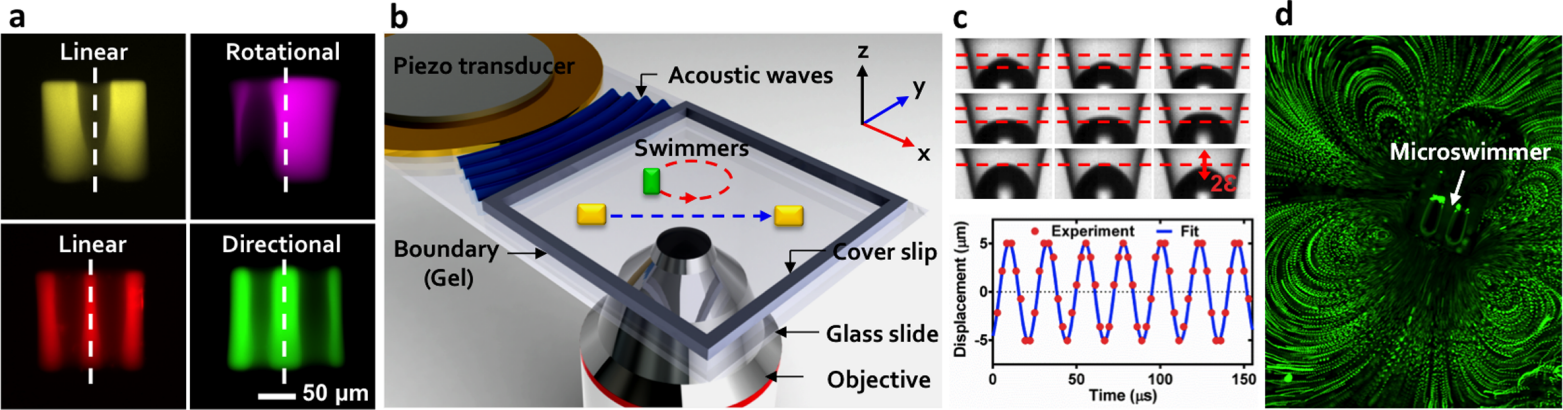
Geometry and experimental design of the acoustic microswimmers. (a) Fluorescent images of four types of swimmer: linear microswimmers with a single (false-coloured yellow) or double (red) indent that is symmetric about the central axis, rotational microswimmers with off-centred (purple) indent and directional microswimmers with (green) indents of different diameter. (b), A piezoelectric transducer injects acoustic energy into a chamber that is filled with fluid, lined with acoustically-absorbent putty, and enclosed on top and bottom by glass slides. (c), An image sequence recorded at 360,000 frames per second showing bubble oscillation within the conical indentation, fitted to a sine function. (d), Acoustic oscillation of the microswimmer bubbles generates substantial acoustic microstreaming in water. Both ends of indentations are open.

The acoustic microswimmer consists of a rectangular polymer body with one or more conical indentations, as shown in Fig. 2a. When the microswimmer is submerged in the liquid-filled chamber, an air bubble can spontaneously become trapped in each of its indentations. A piezoelectric transducer mounted to a glass slide adjacent to the chamber generates the acoustic field, as shown in Fig. 2b (see experimental details in the Methods section). The acoustic cell is designed with absorbing walls to define a predominately traveling-wave acoustic field and avoid complications from the complex nodal structures of standing waves. When the trapped bubble is exposed to an acoustic field with a wavelength much larger than bubble diameter, its resulting oscillations induce a steady flow field around itself at a length scale comparable to bubble size. These oscillations are tracked photographically in Fig. 2c, and the resulting flow field is shown in Fig. 2d. When the frequency of the function generator driving the transducer approaches a resonance of the trapped bubble, the oscillation amplitude of the liquid-air interface reaches a maximum. We exploit this phenomenon to achieve addressable self-propulsion.

**Figure 3:**
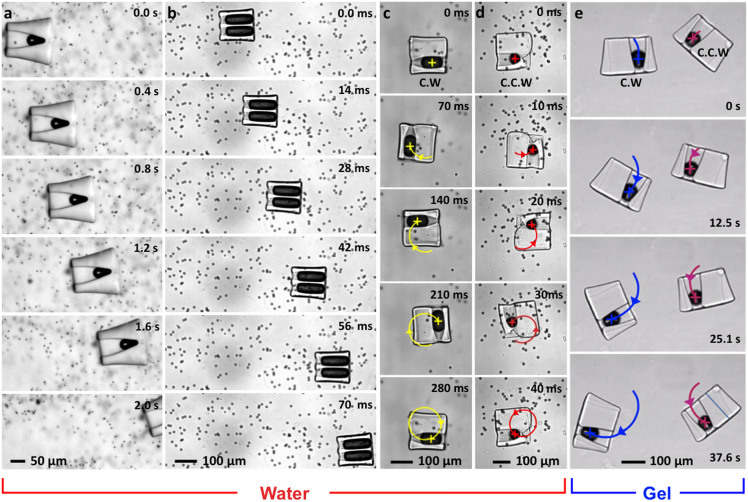
High-speed imaging captures the translational and rotation motion of acoustic microswimmers moving through either a water/microbead mixture or hydrogel. (a), A single on-centre bubble generates linear motion in water, as does (b), a pair of bubbles of equal size symmetrically placed. An off-centre indentation generates either (c), clockwise or (d), counterclockwise motion. (e), The same rotary motion (or linear motion, not shown) can also be achieved in viscous shear-thinning hydrogel.

Bubble size and shape determine the resonance frequency; we control these by changing the indentation diameter (from 50 to 100 microns), the indentation depth (from 70 microns to the full length of the swimmer), and the duration of the hydrophobic treatment. The symmetry of the bubble position(s) within the microswimmer determines the type of motion, as shown in Fig. 2a. One or two symmetric indentations yield the translational motion shown in Fig. 3a,b and Supplementary Videos 1 and 2. Asymmetric or off-centre indentations produce the rotational motion of Fig. 3c,d,e and Supplementary Videos 3, 4, and 5. The exposed bubble surface at the interface with the fluid is important for propulsion and is independent of the shape (conical or cylindrical) of the bubble trapped. The motive force and moment (*i.e.*, torque) created by an acoustically-driven bubble is determined by the intensity of the ambient acoustic field, which is controlled by adjusting the voltage applied to the piezoelectric transducer. The propulsive force or moment can be made large enough to achieve very high translational velocities: up to ~ 8 mm/sec, which is ~ 50 body lengths per second. Rotational velocities likewise can be as large as ~ 20 rotations per second in water. The swimmer speed drops three orders of magnitude in 50% glycerol/water, and speed in viscous hydrogel (discussed later) is much lower but still substantial: 50 µm/s or 3 rotations per minute. The acoustic microswimmer rapidly attains its steady-state speed (see Supplementary Information 1).

In the classic paper “Life at low Reynolds number”, Purcell proposed the scallop theorem: reciprocal motion of a swimmer submerged in fluid at low Reynolds number yields no net motion through the fluid^1^. In accord with this theorem, natural microswimmers such as sperm or bacteria propel themselves by means of non-reciprocal motions of flagella, cilia, or other appendages. Artificial microswimmers inspired by biology have likewise typically sought to achieve non-reciprocal motion. We pursue a different strategy here: while our acoustically driven microbubble swimmer does move *as a whole* at relatively low Reynolds number, the reciprocal oscillations *within its bubble engine* work at moderately high Reynolds number (9 < Re < 90 for 0.5 μm < ε < 4 μm) and exploit the nonlinear inertia of fluid dynamics from high-frequency ultrasound. The applied acoustic wave has a wavelength on the order of a centimetre, two orders of magnitude larger than the microswimmer, thus the swimmer is subject to nearly uniform fluid pressure on all sides. This uniform acoustic environment is confirmed by the lack of motion in bubble-free microswimmers that are immersed in an acoustic field of varying frequency and amplitude (see Supplementary Information 2).

We begin with a high-level discussion of the different potential contributions to the acoustic propulsion^40^ in the physical regime of the acoustic microswimmer, so that we can extract the critical scaling relations that will enable quantitative empirical analysis. The fundamental fluid mechanics fields, density *ρ*, pressure *p*, and velocity *u* all have incident, scattered, and streaming components, where the streaming contribution is defined at the zero-frequency component^41^,^42^,^43^. Acoustic propulsion can arise from either acoustic microstreaming^44^,^45^,^46^,^47^,^48^,^49^,^50^,^51^,^52^ or radiation pressure, since both can carry momentum to infinity. The stress tensor T_jj_ = *pδ_ij_* + *ρu_i_u_j_* can be averaged over the period of oscillation to yield 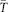, which can then be decomposed into second-order radiative and microstreaming contributions arising from combinations of incident *(i)* and scattered *(s)* fields: 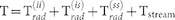. The propulsive force corresponds to the integral of the divergence of this stress tensor over the region surrounding the microswimmer, out to a surface *S*_∞_ located at infinity (and remembering that the stress tensor associated with the incident plane-wave excitation is divergence-free). Taking 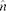 to be an outward normal (*i.e.*, pointing away from the microswimmer), we obtain the propulsive force 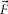:
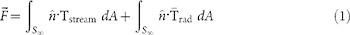
The first term here arises from acoustic microstreaming: this effect has been considered before in the context of pinned bubbles on substrates, as discussed below. The second term arises from the radiative momentum flux. It has two contributions, one from the scattered field along and the other with contributions from both incident and scattered fields:
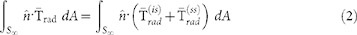
The radiative propulsive force will be proportional to the square of the amplitude of the acoustic field (s). Although the *(is)* and *(ss)* terms have different contributions from incident and scattered waves, the fact that the scattered wave is a linear response to the incident wave implies that the overall radiation-derived acoustic force at fixed frequency for a given bubble is effectively proportional to the square of the observed amplitude of the bubble oscillation in both cases. The underlying linear dependence of the *(is)* term on bubble amplitude could be revealed by holding the incident wave amplitude fixed and instead sweeping its frequency across the resonant response peak of the bubble. Since the incident wave has larger amplitude, it is reasonable to suppose that that the *(is)* term dominates. Since the *(is)* term is essentially an interference term between the incident plane wave and the scattered wave, this contribution to the propulsion should vary depending on the orientation of the microswimmer with respect to the wavevector of the incident plane-wave acoustic excitation. This contribution is also dependent on the acoustic excitation having some degree of standing wave character, since the time average of the *(is)* term would be zero for a pure traveling wave passing over a microswimmer that is much smaller than the acoustic wavelength.

We now turn our attention to the acoustic microstreaming contribution to propulsion. For purposes of defining an axis of streaming, the bubble must be embedded within an asymmetric acoustic environment. The simplest such asymmetry that one can consider is a superposition of spherically symmetric oscillations of the bubble radius of amplitude *ε* plus transverse oscillations of the bubble centre up and down along a given axis of amplitude *ε*. Longuet-Higgins showed that a spherical bubble in an unbounded Newtonian fluid engaged in a superposition of radial and transverse oscillations at frequency *ω* produces a second-order steady flow that scales as *εε* and is linear in *ω*^53^. If the ratio of the radial and transverse oscillations is fixed, then can be simplified to *u* ∝ *ε*^2^*ω*. Marmottant and Hilgenfeldt extended this result to a bubble oscillating near a wall, finding a toroidal steady flow whose symmetry can be broken by a nearby structural asymmetry to yield net fluid flow^54^,^55^. The quadratic scaling in *ε* and linear scaling in *ω* are preserved in these lower-symmetry situations, and similarly should be preserved in our more complex microswimmer geometry, for example, the bubble exposed to fluid on only one side within an indentation. For such a trapped bubble, oscillating with amplitude *ε*, the flow field around the bubble is *u* = *εu*_1_ + *ε*^2^*u*_2_ and in a Newtonian fluid of density *ρ*, the oscillatory first-order *u*_1_induces a second-order steady flow ⟨*u*_2_⟩, governed by an Stokes equation with body force ⟨−*ρu*_1_·∇*u*_1_⟩, which is nonlinear in *u*_1_. For completeness, we also note that in certain cases, the scattered wave can dissipate so rapidly in space that substantial momentum is directly deposited into the ambient fluid to create a radiatively driven streaming flow known as a “quartz wind”. However, in aqueous solution this dissipative mechanism is generally significant only at much higher frequencies^56^ than those (kHz) used in this work.

**Figure 4:**
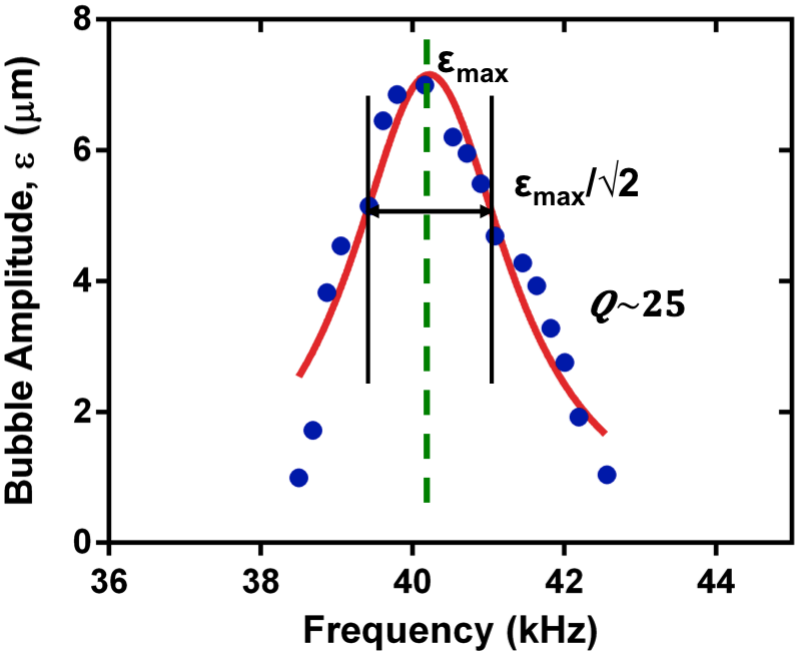
Frequency dependence of bubble oscillation amplitude. The bubble oscillation is largest when the acoustic driving field is resonant with the fundamental natural frequency of the bubble. The resonance peak for a bubble of diameter 45 µm in water is reasonably narrow. Corresponding to a quality factor Q ~ 25.

**Figure 5:**
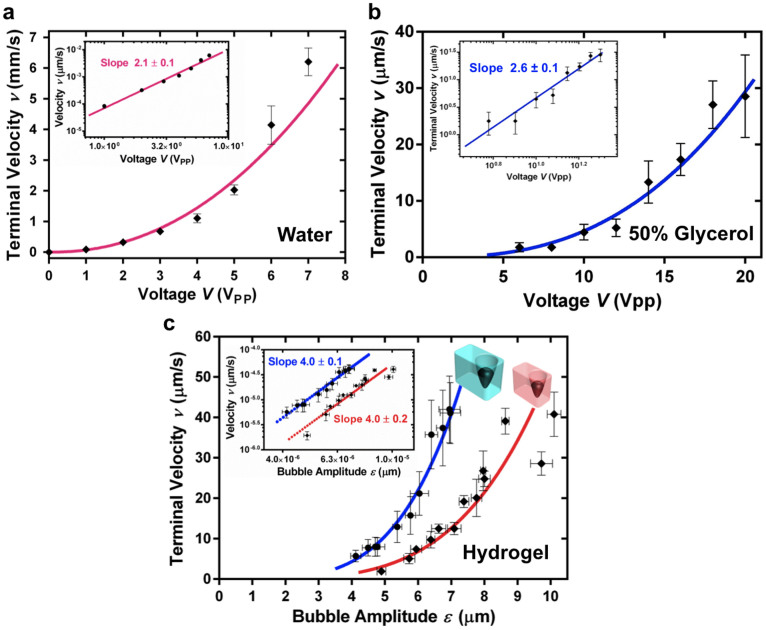
Characterization of the acoustic microswimmers. (a), An acoustic microswimmer immersed in water moves at a speed nearly proportional to the square of the amplitude of the drive voltage, *i.e.*, the square of the amplitude of the incident acoustic field. This dependence is consistent with the acoustic coupling to motility. (b), An acoustic mciroswimmer immersed in a more viscous solution, 50% glycerol, exhibits similar scaling, with a slightly higher slope. (c), Within the shear-thinning hydrogel, the microswimmer speed varies as the fourth power of the bubble oscillation amplitude (measured by direct high-speed imaging). This result is consistent with the shear-thinning behaviour and an acoustic propulsion that scales with the square of the oscillation amplitude. Similar results are obtained for two swimmers with bubble diameters of 30 µm (driven at 94.4 kHz, in red) and 67 µm (driven at 70.4 kHz, in blue).

The resonant response of the bubble oscillation amplitude as a function of frequency is mapped out in Fig. 4 with ±1 μm accuracy. The resonance is reasonably sharp and for typical excitation amplitudes a swimmer that begins at rest will move only when excited close to resonance. For air bubbles in water, viscous damping in the form of acoustic microstreaming dominates over radiation and thermal damping^41^. Using direct high-speed measurements of the fluid/bubble interface, we observed that the amplitude of the bubble oscillation is linearly proportional to the amplitude *V_0_* of the voltage applied to the signal generator in water (see Supplementary Information 3). Thus for a given bubble configuration and at a fixed excitation frequency, the acoustic microstreaming, and hence swimmer speed derived from bubble oscillations, should scale as *V_0_*^*2*^ in water, irrespective of whether the force originates in microstreaming or radiation. Fig. 5a shows that this quadratic relation is reasonably well satisfied by the bubble-powered microswimmer moving in water; the slight deviation from a precise quadratic relation is not surprising, considering that the centre of mass motion of the microswimmer in water is at the edge of the low Reynolds number regime *Re* ≤ 1 (see Supplementary Information 4) and the microswimmer carries a complex acoustic streaming flow pattern around it. The behaviour in 50% glycerol solution (Fig. 5b) is also reasonably close to the anticipated quadratic behaviour.

Since the scaling relation between speed and applied voltage does not distinguish the two motive mechanisms, is there any alternative means to establish whether one or both of these mechanisms is operating here? Fig. 2d shows a strong acoustic microstreaming field in the vicinity of the microswimmer, with a size comparable to that of the bubble itself and microstreaming speed faster than the centre of mass speed of the swimmer. This robust microstreaming pattern strongly suggests that there is net momentum flow and a significant microstreaming-derived acoustic force (see Supplementary Video 8). In addition, there is suggestive but not definitive evidence that the radiation force is significant: the speed of the microswimmer varies significantly (by ~ 25%) as a function of the phase in the circular orbit of an asymmetric microswimmer^37^ (see Supplementary Information 5). Since acoustic microstreaming depends only on the amplitude of the bubble oscillation and not the wave vector of the incident wave, this variation in speed around the orbit suggests that the radiation force derived from the interference of incident and scatter fields may also be significant (with a caveat that acoustic shadowing or residual standing-wave components of the acoustic field could also be involved). An ability to access a regime in which both of these forces are in fact significant would provide additional flexibility in tuning microswimmer properties.

### Experimental demonstration of the acoustic microswimmer in viscous fluids

Although the microswimmer is small, the powerful bubble engine propels it sufficiently quickly that its Reynolds number is comparable to one. To generate centre-of-mass motion at lower Reynolds number and simultaneously reduce the spatial extent of the microstreaming pattern surrounding the microswimmer, we studied behaviour of microswimmers in both 50% glycerol solution and viscous shear-thinning hydrogel. The acoustic microstreaming pattern in these more viscous media is highly localized near the bubble (see Supplementary Information 6). In glycerol solution we obtain *Re* ≤ 10^−2^ and in hydrogel *Re* ~ 10^−6^ (see Supplementary Information 4). These low values ensure that the centre of mass motion occurs at low Reynolds number, although additional complications arise at the bubble/fluid interface in the case of the hydrogel, since it is shear-thinning. Quantification of drag in a shear-thinning fluid is a complex problem much effort has been focused on obtaining approximate expressions in a form similar to the standard Stokes formula, often within a power law model for the variation in viscosity as a function of shear rate *τ* = *K*(∂*u*/∂*y*)*^n^*, where for the hydrogel used K ≈ 9.2 is the flow consistency index and *n* ≈ 0.49 is the flow behaviour index. If we assume a Stokes-like drag expression with a constant correction factor of order unity, then the terminal velocity of the microswimmer in hydrogel should vary as the fourth power of the bubble amplitude. Fig. 5c demonstrates good agreement to this power law for microswimmers with bubbles of two different sizes. This analysis, coupled to estimates of typical drag forces, suggests that the acoustic microswimmer operating in hydrogel generates forces in the microNewton range for typical bubble oscillation amplitudes of several microns (see Supplementary Figure S5).

### Selective addressability of the acoustic microswimmers

**Figure 6:**
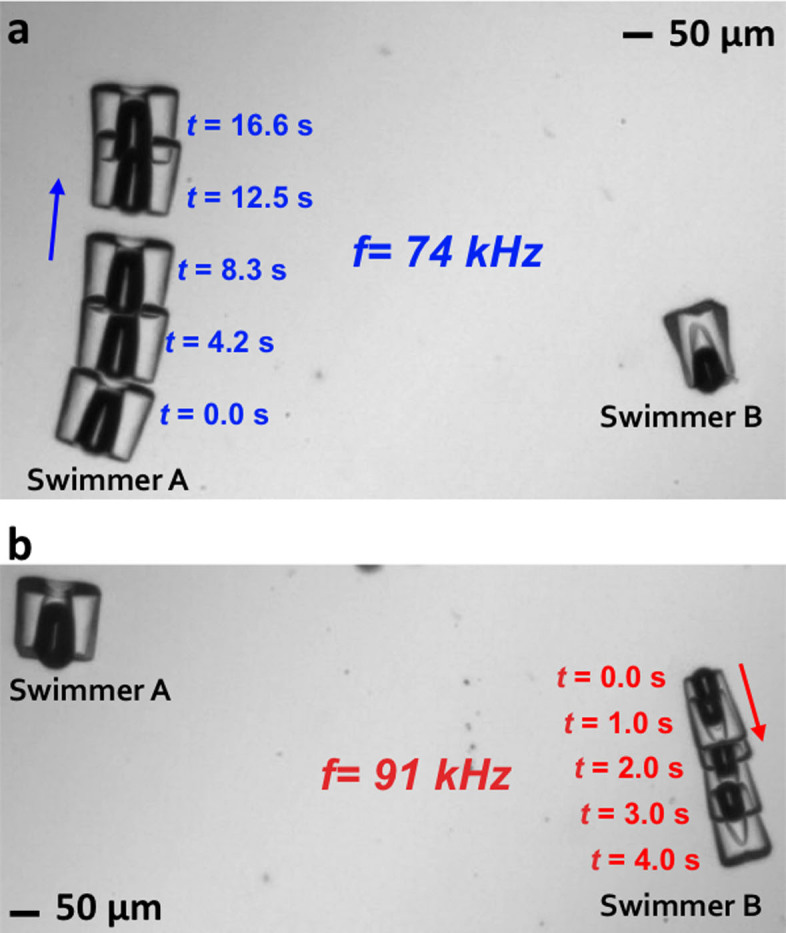
Superimposed time-lapse images of selective actuation of an acoustic microswimmer from within a group. Two swimmers with bubbles of different size were immersed in an acoustic field of variable frequency. (a), Swimmer A, with the larger bubble, begins acoustically-driven motion at 74 kHz, with little simultaneous motion of swimmer B. A video of this behaviour is available as Supplementary Video 6. (b), With further increase in frequency, swimmer A stops; swimmer B begins to move at 91 kHz, with swimmer A remaining essentially stationary. A video of this behaviour is available as Supplementary Video 7.

A key advantage of this resonant acoustic mechanism of propulsion is the ability to selectively address one microswimmer within a group. The quality factor of the bubble resonance is reasonably high, on the order of 25 (see Fig. 4), so that even a small difference in bubble diameter will yield a robust separation in frequency response between different swimmers or possible different bubbles within the same swimmer. To demonstrate this, we fabricated two single-bubble microswimmers with bubbles of different radii and swept the acoustic drive frequency upwards. When the large bubble reached resonance, the frequency was temporarily held fixed and this swimmer translated as shown in Fig. 6a and Supplementary Video 6. A further ramp of the drive frequency reached the resonance of the smaller bubble: the original swimmer stopped and newly resonant swimmer began moving, as shown in Fig. 6b and Supplementary Video 7. Considering the sharpness of the resonant response, it should be possible to selectively address individual microswimmers within groups of multiple microswimmers.

**Figure 7:**
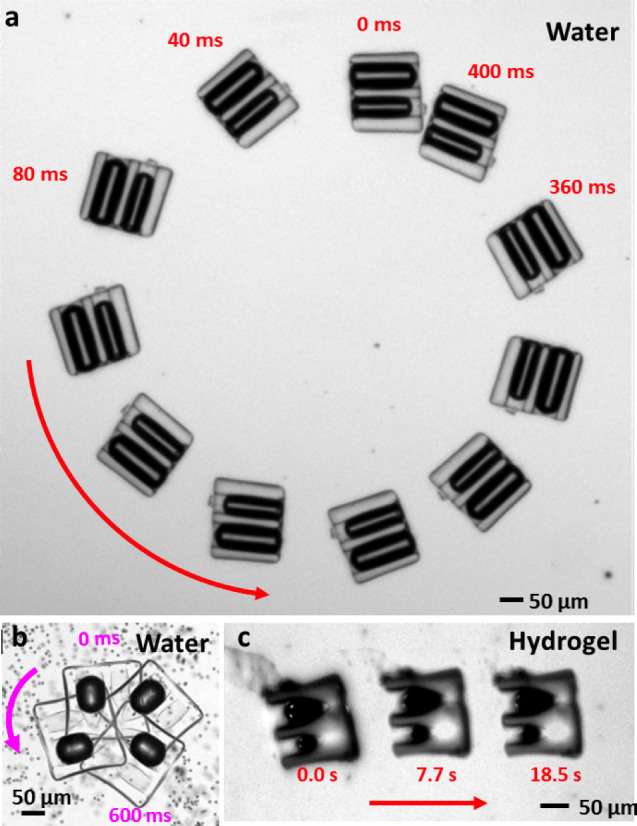
Superimposed time-lapse images of controlled two-dimensional motion of different microswimmers with bubbles of different sizes. (a), When a two-bubble swimmer is driven at the resonance of just one bubble, it rotates in a wide orbit. (b), The orbit of an asymmetric one-bubble swimmer is much tighter due to its stronger asymmetry. (c), At a frequency intermediate between the resonances of the two constituent bubbles, a two-bubble swimmer can move in a straight line.

Unequal frequency-dependent excitation of different bubbles within a multi-bubble swimmer should modulate the degree of translational and rotational motion; ultimately, this could enable two-bubble microswimmers that are fully steerable in two dimensions. We have fabricated microswimmers with two bubbles of different sizes. When the acoustic field excites one bubble more than another, the applied torque leads the swimmer to perform rotational motion as shown in Fig. 7a. The orbital radius of the swimmer is larger than that of a microswimmer with a single off-centre bubble, shown in Fig. 7b, due to some combination of the finite width of the resonances (*i.e.*, the resonances of the two bubbles overlap in frequency) and nonlinear coupling between the bubbles. At this length scale, the effect of stochastic orientational diffusion is negligible and the swimmer follows a nearly perfect circular trajectory with negligible long-run translational diffusion^57^,^58^. When a different two-bubble swimmer is driven at a frequency that equally excites both bubbles, it performs linear translation, as shown in Fig. 7c.

### Tunable Swimmer-swimmer interaction

**Figure 8:**
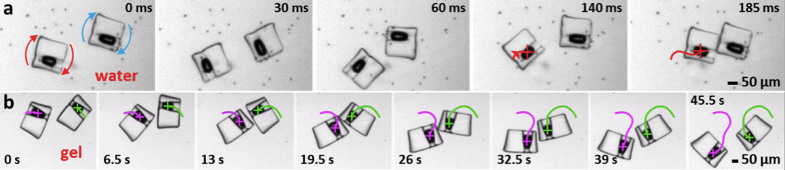
Interaction of two swimmers in water and hydrogel. (a), Two swimmers undergoing clockwise motions in water firmly lock themselves together after snapping into contact, as shown in Supplementary Video 10. (b), Two swimmers in hydrogel, a medium that suppresses acoustic streaming, come apart after collision. The swimmer trajectories across the collision are traced in magenta and green. This event is also shown in Supplementary Video 11.

Tunable swimmer-swimmer interactions are possible: nearby acoustic microswimmers in water can snap into persistent contact whereas similar swimmers in hydrogel collide and separate. Motion may arise due to overlap of acoustic microstreaming fields or the interaction of re-radiated acoustic waves from nearby swimmers (*i.e.*, the secondary Bjerknes force)^59^. To demonstrate this phenomenon, we placed two identical rotational swimmers in water in close vicinity. Under acoustic excitation, the swimmers rotate and drift together and eventually come into contact, as shown in Fig. 8a. Thereafter, the swimmers remain in direct contact. The concept has great potential in collective behaviour; the interactions of multiple swimmers can be controlled by the applied acoustic power. In contrast, two rotational swimmers in viscous hydrogel come apart after collide, as shown in Fig. 8b. In viscous hydrogel, the acoustic waves from the swimmers were attenuated more by absorption when compared to that of water^3^. The lack of sustained contact suggests that significant acoustic microstreaming fields on the order of the swimmer dimensions are important for swimmer-swimmer interactions.

## Discussion

These acoustically powered microswimmers achieve significant advances in performance. The acoustic field is inexhaustible and largely unaffected by the ambient chemical state, unlike mechanisms of chemically powered motility. Biologically benign low-power acoustic fields^60^ can generate sufficient force to propel swimmers through highly viscous fluids, which might be found inside biological systems such as human vasculature. In contrast, electric and magnetic actuation mechanisms often require large, biologically-damaging fields to achieve adequate propulsion. In addition to this excellent performance on conventional metrics, our design also achieves selective actuation of a single swimmer from among a group—a first in the field. Selective actuation opens new possibilities for coherent cooperative action within groups of microswimmers. With a third bubble, steering in three dimensions should be possible. Due to the resonant nature of the bubble in acoustic fields, multiple bubbles of dissimilar resonances can be contained in a single microswimmer at different planes. By selectively actuating each bubble at resonance, motion in different directions could be obtained. In addition, tunable swimmer-swimmer interactions are possible. A collection of selectively actuated steerable microswimmers, their bubble surfaces stabilized by a polymeric coating, could be deployed in vasculature with actuation provided by an exogenous transducer applied to the skin, the resulting behaviour being tracked by ultrasonic or magnetic resonance imaging.

## Methods

### Materials

The microswimmers were fabricated using a mixture of photo-crosslinkable polyethylene glycol (PEG) and a photo-initiator. The mixture consisted of 40% (v/v) PEG diacrylate with a molecular weight of 700 (PEG700, from Sigma-Aldrich), 25% (v/v) PEG with a molecular weight of 258 (PEG 258, from Sigma-Aldrich), 15% (v/v) photo-initiator 2-Hydroxy-2-methyl-1-phenyl-propan-1-one (Darocur 1173, from Ciba), 15% (v/v) TE buffer (100 TE, from OmniPur), and 5% (w/v) fluorescein.

### Swimmer release

After UV exposure selectively hardened the liquid PEG polymer, the hardened polymer bodies of the microswimmers had to be separated from the surrounding liquid polymer. To accomplish this, the swimmers were washed three times in ethanol solution containing 0.05% Tween 20 (from Sigma Aldrich) to remove any liquid PEG residue from the hardened PEG surfaces (including from the indentation).

### Trapping of the bubble

A drop of ethanol solution containing the microswimmers was placed onto the glass slide used for observations under the microscope. This glass slide was heated for 30 min at 65°C to dehydrate the swimmers. The slide was then moved to a vacuum chamber, where the swimmers were treated with 1H, 1H, 2H, 2H-perfluorooctyl-trichlorosilane for 20−30 minutes to make their surfaces hydrophobic. After this hydrophobicity treatment, drops of liquid (water, 50% glycerol solution, or viscous hydrogel) were added to the microswimmers, causing air bubbles to be trapped in the swimmer indentations. The size of the trapped air bubble was a function of the indentation diameter and depth and the hydrophobicity treatment duration.

### Apparatus for microswimmer characterization

The glass platform holding the microswimmers in ambient liquid (see Section 1.4) was either a rectangular glass slide (6.08 cm × 2.54 cm) or a circular petri dish (9 or 18 cm diameter). The liquid was bounded on the perimeter by acoustically absorbent putty and on the top surface by a glass cover slip. Acoustic waves were introduced to the liquid via the glass slide/petri dish, to which was bonded a piezoelectric transducer driven by a function generator (Tektronix AFG 3011). The glass slide/petri dish was mounted on the stage of a Nikon TE-2000U optical microscope.

### Imaging and tracking

Microswimmer motion was captured using a Photron SA4 fast camera connected to the microscope. Raw high-speed images were analysed using NIS tracking software to determine parameters such as translational/rotational velocity.

## Author Contributions

D.A. conceived the initial idea and contributed to the experimental design and performed the bulk of the experimental work, data analysis and interpretation. M.L. contributed substantially to the experimental work, data analysis, and interpretation and helped with the theoretical development. A.N. and P.E.L. were the primary contributors to the theoretical discussion and helped with data analysis. A.N., D.A., Z.S., M.L. and H.S.M. contributed to the writing of the manuscript. V.H.C. and T.J.H. provided overall guidance and contributed to the experimental design and scientific presentation.

## Additional Information

**How to cite this article**: Ahmed, D. *et al* . Selectively manipulable acoustic-powered microswimmers. *Sci. Rep.* **5**, 9744; doi: 10.1038/srep09744 (2015).

## Supplementary Material

Supplementary Information Supplementary Information

Supplementary Information Supplementary Video 1

Supplementary Information Supplementary Video 2

Supplementary Information Supplementary Video 3

Supplementary Information Supplementary Video 4

Supplementary Information Supplementary Video 5

Supplementary Information Supplementary Video 6

Supplementary Information Supplementary Video 7

Supplementary Information Supplementary Video 8

Supplementary Information Supplementary Video 9

Supplementary Information Supplementary Video 10

Supplementary Information Supplementary Video 11

## References

[b1] Purcell E. M. Life at low Reynolds number. Am. J. Phys. 45, 3 (1977).

[b2] Nawroth J. C. *et al.* A tissue-engineered jellyfish with biomimetic propulsion. Nat. Biotechnol. 30, 792–7 (2012). 22820316 10.1038/nbt.2269 PMC4026938

[b3] Won J. M., Lee J. H., Lee K. H., Rhee K. & Chung S. K. Propulsion of water-floating objects by acoustically oscillating microbubbles. Int. J. Precis. Eng. Manuf. 12, 577–580 (2011).

[b4] Patra D. *et al.* Intelligent, self-powered, drug delivery systems. Nanoscale 5, 1273–83 (2013). 23166050 10.1039/c2nr32600k

[b5] Gourevich D. *et al.* Ultrasound-mediated targeted drug delivery with a novel cyclodextrin-based drug carrier by mechanical and thermal mechanisms. J. Control. release 170, 316–24 (2013). 23770006 10.1016/j.jconrel.2013.05.038

[b6] Nelson B. J., Kaliakatsos I. K. & Abbott J. J. Microrobots for minimally invasive medicine. Annu. Rev. Biomed. Eng. 12, 55–85 (2010). 20415589 10.1146/annurev-bioeng-010510-103409

[b7] Mirkovic T., Zacharia N. S., Scholes G. D. & Ozin G. A. Fuel for thought: chemically powered nanomotors out-swim nature's flagellated bacteria. ACS Nano 4, 1782–9 (2010). 20420469 10.1021/nn100669h

[b8] Sacanna S. *et al.* Shaping colloids for self-assembly. Nat. Commun. 4, 1688 (2013). 23575692 10.1038/ncomms2694

[b9] Palacci J., Sacanna S., Steinberg A. P., Pine D. J. & Chaikin P. M. Living crystals of light-activated colloidal surfers. Science 339, 936–40 (2013). 23371555 10.1126/science.1230020

[b10] Chronis N. & Lee L. P. Electrothermally activated SU-8 microgripper for single cell manipulation in solution. J. Microelectromechanical Syst. 14, 857–863 (2005).

[b11] Junkin M., Leung S. L., Whitman S., Gregorio C. C. & Wong P. K. Cellular self-organization by autocatalytic alignment feedback. J. Cell Sci. 124, 4213–20 (2011). 22193956 10.1242/jcs.088898 PMC3258106

[b12] Liu M., Zentgraf T., Liu Y., Bartal G. & Zhang X. Light-driven nanoscale plasmonic motors. Nat. Nanotechnol. 5, 570–3 (2010). 20601945 10.1038/nnano.2010.128

[b13] Lee T.-C. *et al.* Self-propelling nanomotors in the presence of strong Brownian forces. Nano Lett. 14, 2407–12 (2014). 24707952 10.1021/nl500068n PMC4039130

[b14] Schamel D. *et al.* Nanopropellers and their actuation in complex viscoelastic media. ACS Nano 8, 8794–801 (2014). 24911046 10.1021/nn502360t

[b15] Paxton W. F. *et al.* Catalytic nanomotors: autonomous movement of striped nanorods. J. Am. Chem. Soc. 126, 13424–31 (2004). 15479099 10.1021/ja047697z

[b16] Wang Y. *et al.* Bipolar electrochemical mechanism for the propulsion of catalytic nanomotors in hydrogen peroxide solutions. Langmuir 22, 10451–6 (2006). 17129015 10.1021/la0615950

[b17] Laocharoensuk R., Burdick J. & Wang J. Carbon-nanotube-induced acceleration of catalytic nanomotors. ACS Nano 2, 1069–75 (2008). 19206505 10.1021/nn800154g

[b18] Zacharia N. S., Sadeq Z. S. & Ozin G. A. Enhanced speed of bimetallic nanorod motors by surface roughening. Chem. Commun. 5856–5858. doi:10.1039/B911561G. (2009). 10.1039/b911561g 19787120

[b19] Fattah Z. *et al.* Straightforward single-step generation of microswimmers by bipolar electrochemistry. Electrochim. Acta 56, 10562–10566 (2011).

[b20] Ota S., Wang S., Wang Y., Yin X. & Zhang X. Lipid bilayer-integrated optoelectronic tweezers for nanoparticle manipulations. Nano Lett. 13, 2766–70 (2013). 23659726 10.1021/nl400999f

[b21] Wu J. *et al.* Motion-based DNA detection using catalytic nanomotors. Nat. Commun. 1, 36 (2010). 20975708 10.1038/ncomms1035

[b22] Calvo-Marzal P. *et al.* Electrochemically-triggered motion of catalytic nanomotors. Chem. Commun. 4509–4511. doi:10.1039/B909227G. (2009). 10.1039/b909227g 19617966

[b23] Zhang L. *et al.* Characterizing the swimming properties of artificial bacterial flagella. Nano Lett. 9, 3663–7 (2009). 19824709 10.1021/nl901869j

[b24] Snezhko A., Belkin M., Aranson I. & Kwok W.-K. Self-Assembled Magnetic Surface Swimmers. Phys. Rev. Lett. 102, 118103 (2009). 19392241 10.1103/PhysRevLett.102.118103

[b25] Dreyfus R. *et al.* Microscopic artificial swimmers. Nature 437, 862–5 (2005). 16208366 10.1038/nature04090

[b26] Ghosh A. & Fischer P. Controlled propulsion of artificial magnetic nanostructured propellers. Nano Lett. 9, 2243–5 (2009). 19413293 10.1021/nl900186w

[b27] Sing C. E., Schmid L., Schneider M. F., Franke T. & Alexander-Katz A. Controlled surface-induced flows from the motion of self-assembled colloidal walkers. Proc. Natl. Acad. Sci. U. S. A. 107, 535–40 (2010). 20080716 10.1073/pnas.0906489107 PMC2818967

[b28] Loget G. & Kuhn A. Electric field-induced chemical locomotion of conducting objects. Nat. Commun. 2, 535 (2011). 22086336 10.1038/ncomms1550

[b29] Camacho-Lopez M., Finkelmann H., Palffy-Muhoray P. & Shelley M. Fast liquid-crystal elastomer swims into the dark. Nat. Mater. 3, 307–10 (2004). 15107840 10.1038/nmat1118

[b30] Wang W., Castro L. A., Hoyos M. & Mallouk T. E. Autonomous motion of metallic microrods propelled by ultrasound. ACS Nano 6, 6122–32 (2012). 22631222 10.1021/nn301312z

[b31] Feng J. & Cho S. K. Micro propulsion in liquid by oscillating bubbles. Proceedings of the 2013 IEEE 26th International Conference on Micro Electro Mechanical Systems, Taipei, Taiwan, 20–24 January 2013; pp. 63–66 (2013).

[b32] Dijkink R. J., van der Dennen J. P., Ohl C. D. & Prosperetti A. The ‘acoustic scallop’: a bubble-powered actuator. J. Micromech. Microeng. 16, 1653–1659 (2006).

[b33] Feng J. & Cho S. K. Mini and Micro Propulsion for Medical Swimmers. Micromachines. 5, 97–113 (2014).

[b34] Kao J., Wang X., Warren J., Xu J. & Attinger D. A bubble-powered micro-rotor: conception, manufacturing, assembly and characterization. J. Micromech. Microeng. 17, 2454–2460 (2007).

[b35] Jiang H.-R., Yoshinaga N. & Sano M. Active Motion of a Janus Particle by Self-Thermophoresis in a Defocused Laser Beam. Phys. Rev. Lett. 105, 268302 (2010). 21231718 10.1103/PhysRevLett.105.268302

[b36] Wilson D. A., Nolte R. J. M. & van Hest J. C. M. Autonomous movement of platinum-loaded stomatocytes. Nat. Chem. 4, 268–74 (2012). 22437710 10.1038/nchem.1281

[b37] Fomin V. M. *et al.* Propulsion Mechanism of Catalytic Microjet Engines. IEEE Trans. Robot. 30, 40–48 (2014). 25177214 10.1109/TRO.2013.2283929 PMC4149210

[b38] Ikezoe Y., Washino G., Uemura T., Kitagawa S. & Matsui H. Autonomous motors of a metal-organic framework powered by reorganization of self-assembled peptides at interfaces. Nat. Mater. 11, 1081–5 (2012). 23104155 10.1038/nmat3461 PMC3505225

[b39] Manjare M., Yang B. & Zhao Y.-P. Bubble Driven Quasioscillatory Translational Motion of Catalytic Micromotors. Phys. Rev. Lett. 109, 128305 (2012). 23005998 10.1103/PhysRevLett.109.128305

[b40] Démoré C. E. M. *et al.* Acoustic Tractor Beam. Phys. Rev. Lett. 112, 174302 (2014). 24836252 10.1103/PhysRevLett.112.174302

[b41] Leighton T. G. The acoustic bubble. 129 (Academic Press, 1994).

[b42] Lighthill S. J. Acoustic streaming. J. Sound Vib. 61, 391–418 (1978).

[b43] Ainslie M. A. & Leighton T. G. Review of scattering and extinction cross-sections, damping factors, and resonance frequencies of a spherical gas bubble. J. Acoust. Soc. Am. 130, 3184–208 (2011). 22087992 10.1121/1.3628321

[b44] Wiklund M., Green R. & Ohlin M. Acoustofluidics 14: Applications of acoustic streaming in microfluidic devices. Lab Chip 12, 2438–51 (2012). 22688253 10.1039/c2lc40203c

[b45] Neild A., Rogers P. & Xu L. Particle sorting using an oscillating microbubble. J. Acoust. Soc. Am. 131, 3302–3302 (2012).

[b46] Ding X. *et al.* Surface acoustic wave microfluidics. Lab on a Chip, **13**, pp. 3626–3649 (2013). 23900527 10.1039/c3lc50361e PMC3992948

[b47] Ahmed D. *et al.* Acoustofluidic chemical waveform generator and switch. Anal. Chem. 86, 11803–11810 (2014). 25405550 10.1021/ac5033676 PMC4255676

[b48] Ozcelik A. *et al.* An acoustofluidic micromixer via bubble inception and cavitation from microchannel sidewalls. Anal. Chem. 86, 5083–5088 (2014). 24754496 10.1021/ac5007798 PMC4033639

[b49] Ahmed D. *et al.* Tunable, pulsatile chemical gradient generation via acoustically driven oscillating bubbles. Lab Chip 13, 328–331 (2013). 23254861 10.1039/c2lc40923b PMC3991780

[b50] Ahmed D., Mao X., Shi J., Juluri B. K. & Huang T. J. Sub-milliseconds homogenous mixing using single bubble streaming phenomenon. Lab Chip 9, 2738–2741 (2009). 19704991 10.1039/b903687c

[b51] Ahmed D., Mao X., Juluri B. K. & Huang T. J. Fast microfluidic mixer via acoustic driven sidewall trapped bubbles. Microfluid. and Nanofluid. 7, 727–731 (2009).

[b52] Xie Y. *et al.* Single-shot characterization of enzymatic reaction constants Km and kcat by an acoustic-driven, bubble-based fast micromixer. Anal. Chem. 84, 7495–7501 (2012). 22880882 10.1021/ac301590y PMC3991781

[b53] Longuet-Higgins M. S. Viscous streaming from an oscillating spherical bubble. Proc. R. Soc. A Math. Phys. Eng. Sci. 454, 725–742 (1998).

[b54] Marmottant P. & Hilgenfeldt S. Controlled vesicle deformation and lysis by single oscillating bubbles. Nature 423, 153–6 (2003). 12736680 10.1038/nature01613

[b55] Marmottant P., Raven J. P., Gardeniers H., Bomer J. G. & Hilgenfeldt S. Microfluidics with ultrasound-driven bubbles. J. Fluid Mech. 568, 109 (2006).

[b56] Rife J. *et al.* Miniature valveless ultrasonic pumps and mixers. Sensors Actuators A Phys. 86, 135–140 (2000).

[b57] Nourhani A., Byun Y.-M., Lammert P. E., Borhan A. & Crespi V. H. Nanomotor mechanisms and motive force distributions from nanorotor trajectories. Phys. Rev. E 88, 062317 (2013). 10.1103/PhysRevE.88.062317 24483454

[b58] Nourhani A., Lammert P. E., Borhan A. & Crespi V. H. Chiral diffusion of rotary nanomotors. Phys. Rev. E 87, 050301 (2013). 10.1103/PhysRevE.87.050301 23767469

[b59] Yoshida K., Fujikawa T. & Watanabe Y. Experimental investigation on reversal of secondary Bjerknes force between two bubbles in ultrasonic standing wave. J. Acoust. Soc. Am. 130, 135–44 (2011). 21786884 10.1121/1.3592205

[b60] Ding X. *et al.* Cell separation using tilted-angle standing surface acoustic waves. Proc. Natl. Acad. Sci. U. S. A., 111, 12992–12997 (2014). 25157150 10.1073/pnas.1413325111 PMC4246961

