# Supplementary material for: Selectively manipulable acoustic-powered microswimmers
Source: Sci Rep. 2015 May 20;5:9744. doi: 10.1038/srep09744 (PMC4438614; doi:10.1038/srep09744)
Supplement: Supplementary Information [file srep09744-s1.pdf]

Supplementary Information

Selectively manipulable acoustic-powered microswimmers

Daniel Ahmed[1], Mengqian Lu[1], Amir Nourhani[2], Paul E. Lammert[2], Zak Stratton[1], Hari S. Muddana[3], Vincent H. Crespi[2,4,5*] and Tony Jun Huang[1,3*]

[1]*Department of Engineering Science and Mechanics, The Pennsylvania State University, University Park, Pennsylvania 16802, USA*

[2]*Department of Physics,The Pennsylvania State University, University Park, Pennsylvania 16802, USA*

[3]*Department of Bioengineering, The Pennsylvania State University, University Park, Pennsylvania, 16802 USA*

[4]*Department of Materials Science and Engineering, The Pennsylvania State University, University Park, PA 16802, USA*

[5]*Department of Chemistry, The Pennsylvania State University, University Park, Pennsylvania 16802, USA*

* To Whom Correspondence should be Addressed	
**Professor Vincent H. Crespi**<br>Department of Physics<br>The Pennsylvania State University<br>104 Davey Lab<br>University Park, PA 16802 (USA)<br>Tel: (+1)-814- 863- 0163<br>Fax: (+1)-814-865-3604<br>Email:crespi@phys.psu.edu	**Professor Tony Jun Huang**<br>Department of Engineering Science and Mechanics<br>The Pennsylvania State University<br>N-330 Millennium Science Complex<br>University Park, PA 16802 (USA)<br>Tel: (+1)-814-863-4209<br>Fax: (+1)-814-865-9974<br>Email: junhuang@psu.edu

1. Terminal velocity of the microswimmer

The swimmer rapidly reaches terminal velocity with modest fluctuations in speed, as shown below for a single-bubble swimmer moving in gel. The swimmer was initially at rest at t = 0. The initial transient is due to a slight translation of the bubble within the swimmer as the acoustic field is turned on and the bubble seats more firmly inside the conical depression.

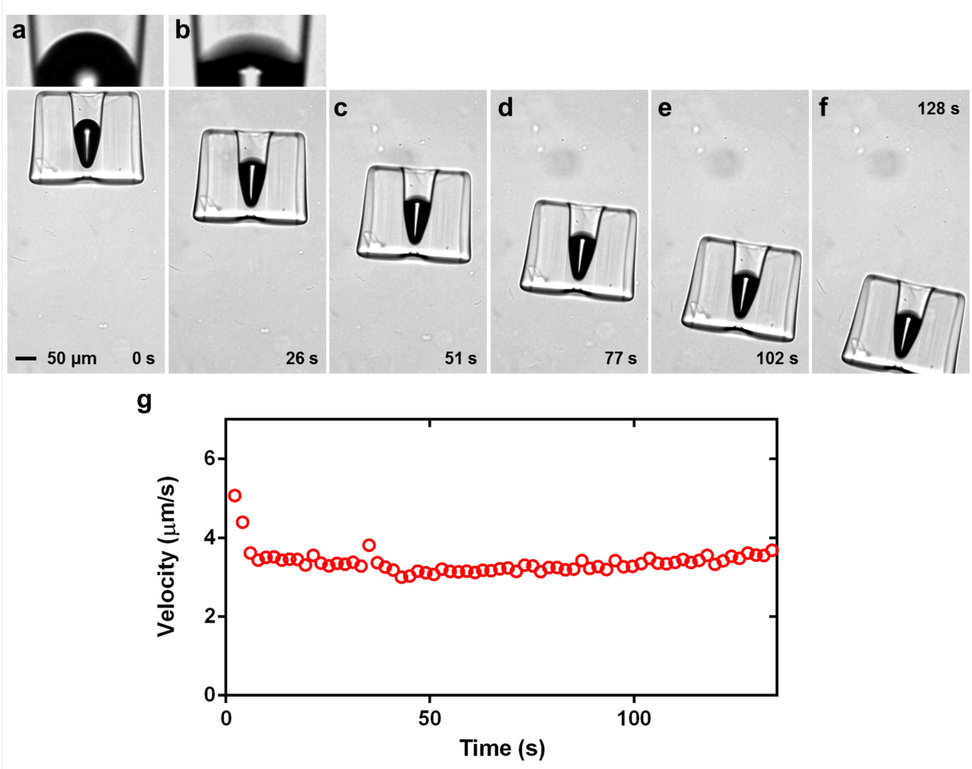

Figure S1: (a–f) Image sequence demonstrates linear motion in a viscous gel (see Supplementary Video 9). **(g)** The swimmer's velocity as a function of time, showing modest fluctuations.

2. Bubble-free swimmers

Control experiments verified that a bubble-less swimmer does not propel. We placed a linear motor (with no trapped bubble) in water/bead solutions shown in Fig. S2(a) then swept the frequency of the acoustic waves from 20 to 120 kHz at the highest voltage applied (20 V_{PP}). No motion was observed. To ensure that adherence of the swimmer to the substrate was not suppressing motility, we placed a second bubble-less motor (this time rotational) within viscous gel as shown in Fig. S2(b). The motor was not in contact with any solid surface. Theacoustic wave was again swept from 20 to 120 kHz at 20 V_{PP} and no motion was observed.

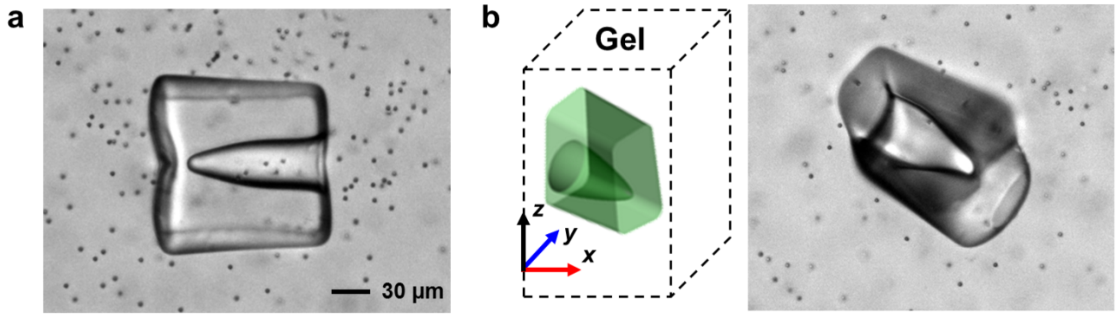

Figure S2:(a) Linear motor with no trapped bubble was placed in water containing bead solutions. **(b)** A rotational motor with no trapped bubble embedded in viscous gel and out of contact with the chamber walls.

3. Relation between voltage and bubble oscillation amplitude

Here we determine the relationship between the bubble amplitude oscillation ε and the amplitude V_0 of the voltage applied to the piezoelectric transducer. Experiments were performed by fixing the microswimmer in place with its bubble driven at resonance. The voltage applied was then increased by one-volt increments and the corresponding oscillation response was recorded by a fast camera. Figure S3 shows the linear relation between the voltage applied and the bubble oscillation amplitude.

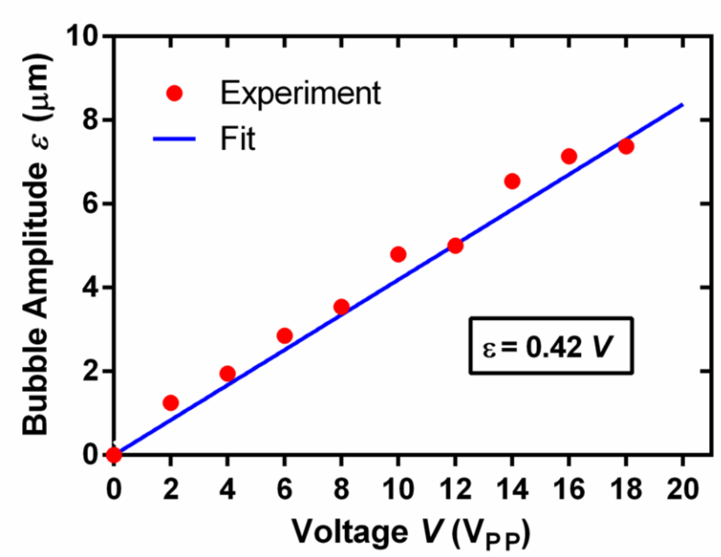

Figure S3: Linear relation between input signal voltage and bubble oscillation amplitude in water (with drive frequency held constant).

4. Reynolds number, drag

In water, the Reynolds number $Re = ud_V/\vartheta$ for the swimmer's centre of mass motion varies from 0.075 to 0.9 for speeds u varying from 500 to 6000 μm/s, where $d_V \approx 150$ μm is the width of the swimmer and $\vartheta \approx 1.0\times10^{-6}$ m^2/s is the kinematic viscosity. In 50% glycerol solution we obtain $Re = 8.0\times10^{-2}$ at maximum speed of $u \approx 30$ μm/s, with $\vartheta \approx 8.3\times10^{-6}$m^2/s. In viscous shear-thinning gel we obtain $Re = 2.1\times10^{-6}$ for the observed maximum speed of $u \approx 50$ μm/s, $d_V \approx 250$ μm, and $\vartheta \approx 0.006$ m^2/s. The oscillating fluid/bubble interface operates at much higher Reynolds number. In water $Re_{interface} = 4\pi af\varepsilon/\vartheta \approx 90$, where a ~ 30 μm is the bubble radius, f = 48 kHz is the excitation frequency, and ε ~ 5 μm is the maximum displacement amplitude. In 50% glycerol solution, $Re_{interface} = 4\pi af\varepsilon/\vartheta \approx 10$, where a ~ 20 μm is the bubble radius, f = 70 kHz is the excitation frequency, and ε~ 5 μm is the maximum amplitude displacement. In viscous hydrogel, $Re_{interface} = 4\pi af\varepsilon/\vartheta \approx 0.02$, where a ~ 20 μm is the bubble radius, f = 70 kHz is the excitation frequency, and ε ~ 7 μm is the maximum amplitude displacement, if we presume $\vartheta \approx 0.006$ m^2/s.

4.1. Drag force acting on microswimmer

The swimmer quickly reaches terminal velocity, at which the propulsive force F_P is counteracted by an equivalent drag force. The drag force acting on the swimmer can be estimated as

$$F_D = 3\pi\mu u d_V C_d,$$

where μ is dynamic viscosity of the liquid, u is the swimmer velocity, d_V (118 μm for small swimmer and 250 μm for big swimmer) is the width of the swimmer, and C_d is the dynamic shape factor. Because the viscous gel exhibits shear thinning, the dynamic viscosity μ in the drag force equation is replaced by an approximate effective viscosity $\mu_e \cong K(u/d_V)^{n-1}$. The power-law model for effective viscosity is simplistic, and a coefficient Y of order of unity is often used to bring model results in line with the observed viscosity ($\mu_e \cong K\left(\frac{u}{d_V}\right)^{n-1} Y$). Thus the drag force counteracting swimmer movement in the gel can be estimated as

$$F_D = 3\pi K u^n {d_V}^{2-n} C_d Y.$$

When the swimmer reaches terminal velocity, $F_P = F_D$. As discussed in the main text, $F_P \propto \varepsilon^2$ or $F_P \propto {V_0}^2$. Therefore, in water or in glycerol solution, the velocity of the microswimmer can be controlled via the input voltage through the relation $u \propto V_0^2$ (with ω held constant). In viscous shear-thinning hydrogel, the flow behaviour index n was measured as 0.49, as shown in Fig. S4. A Plot of drag force versus bubble amplitude for the two swimmers with bubble diameters of 30 µm and 67 µm in the shear thinning hydrogel was shown in Fig. S5. Equating F_P and F_D in the hydrogel (again with ω held constant) yields $u \propto \varepsilon^{4.0}$.

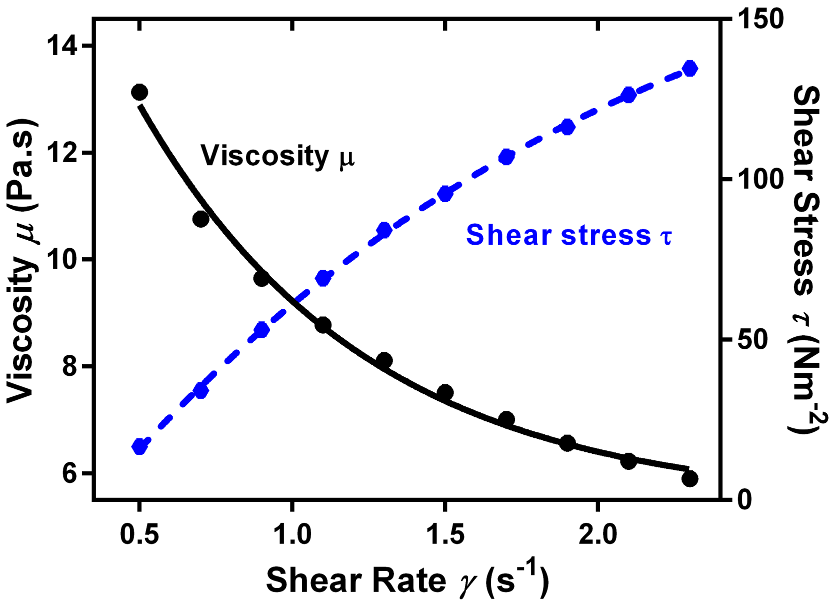

Figure S4: Viscosity μ (solid black) and shear stress τ (dashed blue) of the viscous shear-thinning hydrogel versus shear rate γ. Flow consistency index K and flow behavior index n are experimentally measured as 9.2 and 0.49 for the power-law fluid.

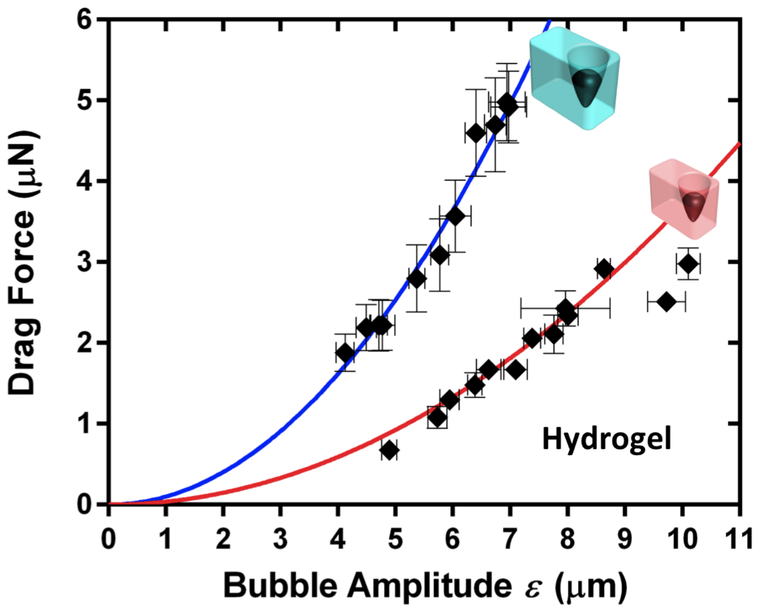

Figure S5: Plot of drag force versus bubble amplitude for two swimmers with bubble diameters of 30 μm and 67 μm in the shear thinning hydrogel.

5. Propulsion variation depending on the orientation

Since the interference term between the incident plane wave and the scattered wave depends on the orientation of the micromotor with respect to the wavevector of the incident plane-wave acoustic excitation, the propulsion should vary during the rotary motion, as shown in Fig. S6.

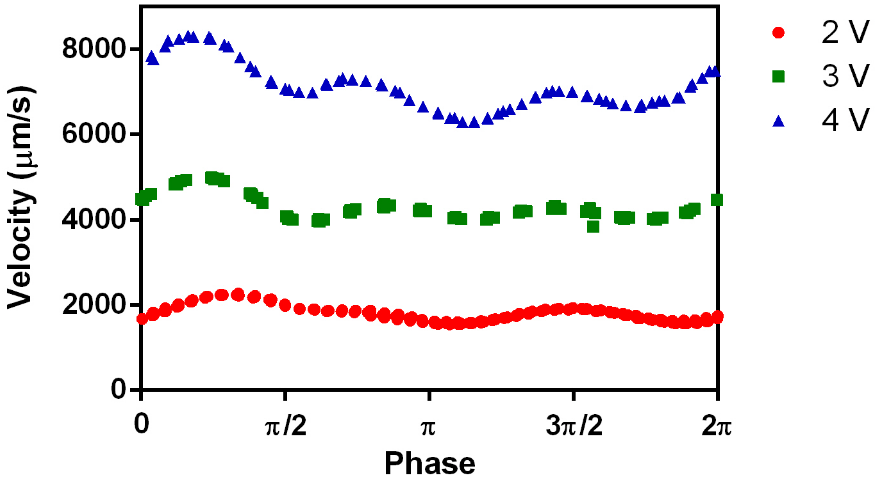

Figure S6: The variation in velocity during the rotary motion in water.

6. Acoustic streaming in water and in gel

When a microswimmer propels in water, acoustic streaming develops in the form of larger counter-rotating vortices. In addition to potentially carrying momentum away from the swimmer as a mechanism of propulsion, these vortices can also affect the hydrodynamic drag on the swimmer. The time-lapse image of Fig. S7(a) shows the strong acoustic streaming in water, the images being obtained when the swimmer is immobilized. In contrast, a swimmer in viscous hydrogel produces no observable acoustic streaming, as shown in Fig.S7(b, c).

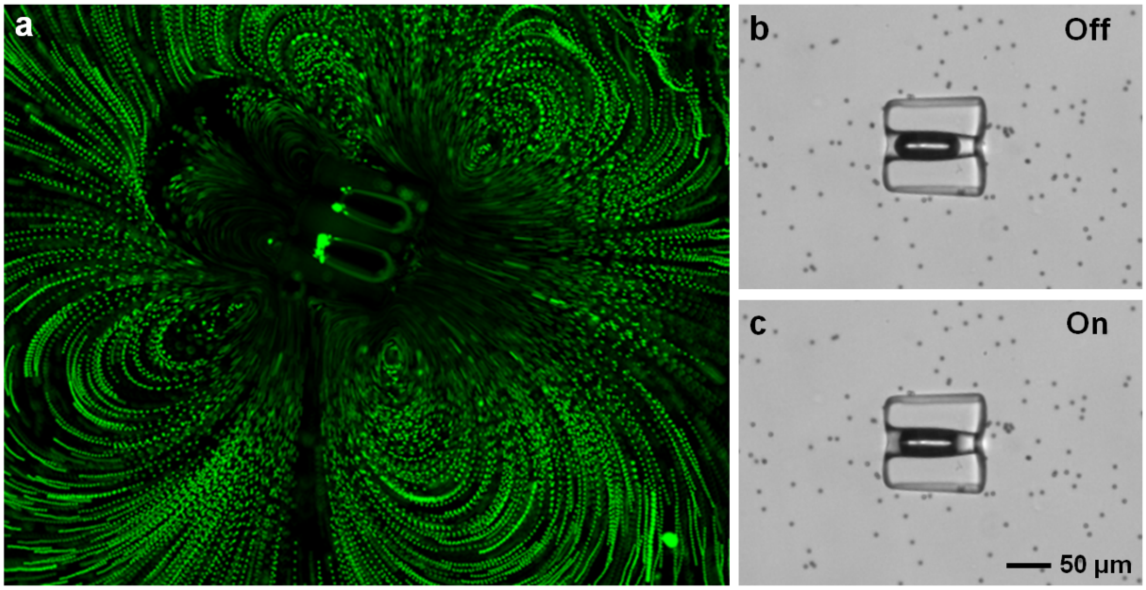

Figure S7: (a) Acoustic driving of the microswimmer bubbles generates substantial acoustic streaming in water (a wider view of Figure 1d from the main text). (**b)** Swimmers immersed in viscous liquid in the absence of acoustic waves. (**c)** The same swimmer in the presence of acoustic excitation: no streaming was observed, only in-place oscillations of 4 μm tracer particles located close to the bubble surface.

Video Legends:

Supplementary Video 1: Linear propulsion of single-bubble swimmer in water

(427 KB)

This video corresponds to the data presented in Figure 3a. A symmetric microswimmer holding a single bubble of ~ 38 μm diameter was driven in water (with suspended 10 μm polystyrene bead tracers) with acoustic excitation at 90 kHz and 8 V peak-to-peak. The video was captured at 16 frames per second and played at 10 frames per second.

Supplementary Video 2: Linear propulsion of paired-bubble swimmer in water

(374 KB)

This video corresponds to the data presented in Figure 3b. A symmetric microswimmer holding a pair of bubbles of ~ 42μm diameter was driven in water (with suspended 15 μm polystyrene bead tracers) with acoustic excitation at 65 kHz and 10 Vpeak-to-peak. The video was captured at 3600 frames per second and played at 10 frames per second.

Supplementary Video 3: Clockwise rotation of an asymmetric swimmer

(4.4 MB)

This video corresponds to the data presented in Fig. 3c. A single asymmetric swimmer holding a bubble of ~ 43μm diameter was driven in water (with suspended 10 μm polystyrene bead tracers) at 57 kHz and 4 Vpeak-to-peak. The video was captured at 10,000 frames per second and played at 24 frames per second.

Supplementary Video 4: Counterclockwise rotation of an asymmetric swimmer

(1.57 MB)

This video corresponds to the data presented in Fig. 3d. A single asymmetric swimmer, holding a ~ 47μm bubble (diameter), opposite to the arrangement shown in Figure 2c,was driven in water (with suspended 15 μm polystyrene bead tracers) at 51 kHz and 7V peak-to-peak. The video is captured at 10,000 frames per second and played at 24 frames per second.

Supplementary Video 5: Rotary motion of swimmers in viscous hydrogel

(1.5 MB)

This video corresponds to the data presented in Fig. 3e. Two identical swimmers, each holding a bubble of ~ 40 μm diameter, were driven in hydrogel at 70.4 kHz and 9 V peak-to-peak. The swimmers rotate in separate orbits with little indication of interaction. The video is captured at 15 frames per second and played at 24 frames per second.

Supplementary Video 6: Autonomous motion of swimmer A

(387 KB)

This video corresponds to the data presented in Fig. 6a. A swimmer with a ~ 40μm diameter bubble is driven in hydrogel at 74 kHz and 15 Vpeak-to-peak. The video is captured at 60 frames per second and played at 24 frames per second.

Supplementary Video 7: Autonomous motion of swimmer B

(115KB)

This video corresponds to the data presented in Fig. 6b. A swimmer with a ~ 30μm diameter bubble is acoustically driven in hydrogel at 91 kHz and 13 V peak-to-peak. The video is captured at 60 frames per second and played at 24 frames per second.

Supplementary Video 8: Visualization of net motion generated by acoustically-driven oscillating bubble

(249 KB)

This video shows a microswimmer fixed in place, with its bubble driven at a resonance frequency. A microparticle in the water, which is small enough that it moves with the water due to drag forces, can be seen to move along the bubble axis. The particle's movement is periodically away from the bubble and then towards the bubble with a longer-term drift away from the bubble.Thus, this video demonstrates the net motion of the water, caused by the reciprocal oscillation of the bubble interface. The video is captured at 360,000 frames per second and played at 24 frames per second.

Supplementary Video 9: Linear motion in viscous gel

(504 KB)

This video corresponds to the data presented in Figure S1. Here, a linear motor is propelled in viscous gel at frequency and voltage 70 kHz and 17 V_{PP} respectively. The video is captured at 15 frames per second and played at 24 frames per second.

Supplementary Video 10: Interaction of multiple swimmers in water

(1.6 MB)

This video corresponds to the data presented in Fig. 8a. Two identical asymmetric swimmers, each holding bubble ~ 46 μm in diameter, were driven in water (with 10 μm suspended polystyrene bead tracers) at 51 kHz and 5 V peak-to-peak. The swimmers begin to interact noticeably at ~ 230 μm separation; they then snap into persistent contact and there after act as a single unit. The video is captured at 10,000 frames per second and played at 50 frames per second.

Supplementary Video 11: "Hit-and-run" of two swimmers in hydrogel

(1.5 MB)

This video corresponds to the data presented in Fig. 8b. Two identical swimmers were driven in hydrogel at 65 kHz and 13 V peak-to-peak. The swimmers collide and then separate and continue on essentially independent paths. The video is captured at 15 frames per second and played at 24 frames per second.

References

1. Beyer, R. T. Radiation pressure—the history of a mislabeled tensor. *J. Acoust. Soc. Am.* **63**, 1025-1030 (1978).
